# Development and validation of a predictive model for the risk of endocervical curettage positivity

**DOI:** 10.3389/fonc.2025.1559087

**Published:** 2025-03-18

**Authors:** Fang Feng, Hui-hui Tuo, Jin-meng Yao, Wei-hong Wang, Feng-lan Guo, Rui-fang An

**Affiliations:** ^1^ Department of Gynecology and Obstetrics, The First Affiliated Hospital of Xi’an Jiaotong University, Xi’an, Shaanxi, China; ^2^ Department of Dermatology, The First Affiliated Hospital of Xi’an Jiaotong University, Xi’an, Shaanxi, China

**Keywords:** endocervical curettage, cervical lesions, prediction model, nomogram, clinical decision-making

## Abstract

**Objective:**

This study aimed to analyze the clinical characteristics of patients undergoing endocervical curettage (ECC), identify factors influencing ECC positivity, and develop a predictive model to assess the risk of positive ECC results. The goal was to assist clinicians in making ECC decisions and reduce missed diagnoses of cervical lesions.

**Methods:**

A retrospective analysis was performed on 953 patients who underwent colposcopically directed biopsy and ECC at the gynecology clinic of the First Affiliated Hospital of Xi’an Jiaotong University between October 2021 and September 2023 due to abnormal screening results. Univariate and multivariate logistic regression analyses were used to identify predictive factors for ECC positivity. An individualized prediction model for ECC positivity risk was developed using R Studio, and the model was subsequently evaluated and validated.

**Results:**

Among the 953 women, the ECC positive rate was 31.48% (300/953). Logistic regression analysis identified age (*P*<0.001), human papillomavirus (HPV) status (*P*<0.01), cytology results (*P*<0.05), acetowhite changes (*P*<0.01), Lugol staining (*P*<0.01), and colposcopic impression (*P*<0.01) as independent predictors of ECC positivity. These factors were incorporated into the prediction model for ECC positivity risk. The area under the receiver operating characteristic curve (AUC) of the model was 0.792 (95% CI:0.760–0.824). The Hosmer-Lemeshow test yielded a *χ^2^* value of 10.489 (*P*=0.2324), and the calibration and clinical decision curves demonstrated that the model exhibited satisfactory calibration and clinical utility.

**Conclusions:**

The clinical prediction model developed in this study demonstrated good discrimination, calibration, and clinical utility. It can be used to evaluate the risk of ECC positivity in patients undergoing colposcopy, reduce missed diagnoses of cervical lesions, and aid clinicians in making ECC decisions.

## 1 Introduction

Cervical cancer is one of the most significant public health issues worldwide, with a particularly high burden in many low-income and middle-income countries (1). In 2022, an estimated 661,021 new cases and 348,189 deaths occurred in the world (2), posing a serious threat to women’s health. With the development and use of the cervical cancer vaccine, along with the standardization and widespread adoption of cervical cancer screening, the incidence of cervical cancer has significantly decreased in developed countries. However, in some developing countries, the lack of early prevention measures and standardized management of cervical cancer has kept the incidence and mortality rates high (3).

Currently, a three-step cervical cancer screening method is used internationally: preliminary screening based on human papillomavirus (HPV) testing and/or cytology, followed by colposcopic examination and histopathological diagnosis. Endocervical curettage (ECC), as part of the colposcopic biopsy, can obtain sufficient lesion tissue from the cervical canal. When the squamocolumnar junction (SCJ) is not fully visualized at colposcopy, ECC may improve the sensitivity of the examination (4). However, ECC is an invasive procedure that can be painful, may cause postoperative complications, and increases the overall cost of colposcopy. As a result, various associations offer differing recommendations regarding its indications (5, 6). The consensus on colposcopic application in China acknowledges the feasibility of ECC when necessary (7), but lacks a clear recommendation. In clinical practice, ECC decisions are often based on physicians’ personal experience rather than objective evidence, potentially leading to both missed and excessive diagnoses and treatments. Therefore, the indications and diagnostic value of ECC for cervical lesions warrant further investigation.

This study aimed to support clinicians’ decision-making regarding ECC by collecting clinical data, analyzing factors influencing ECC positivity, and developing and validating a visual model to predict the likelihood of ECC positivity.

## 2 Materials and methods

### 2.1 Population screening

**Figure 1:**
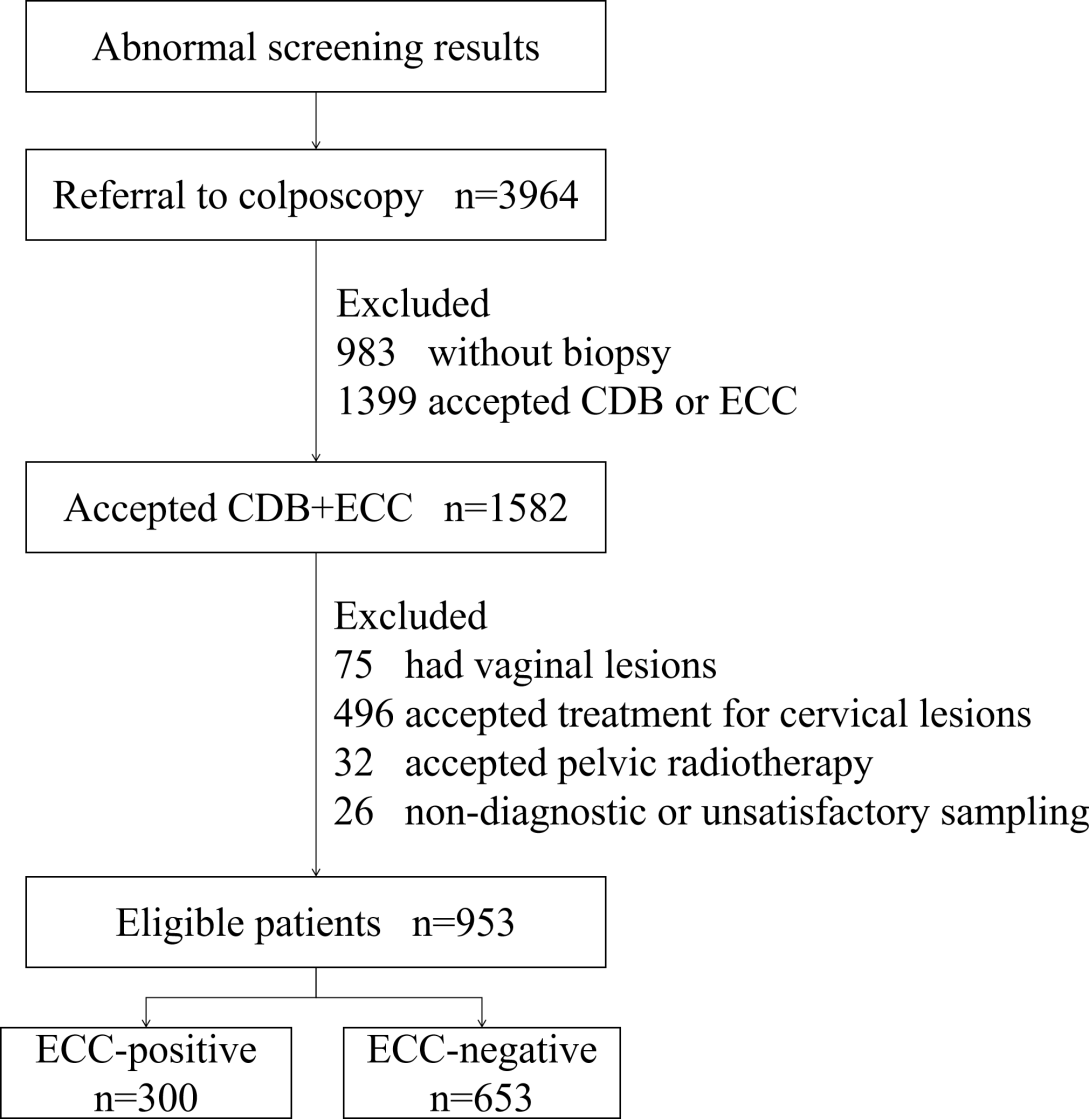
The flow chart of the study.

From October 2021 to September 2023, a total of 953 patients from gynecology clinics were enrolled in this study. They underwent colposcopically directed biopsy (CDB) and ECC during the same period due to abnormal screening results (HPV testing and/or cytology) in the Department of Obstetrics and Gynecology of the First Affiliated Hospital of Xi’an Jiaotong University, Shaanxi Province, China. The inclusion criteria were as follows: participants who (1) had a sexual history; (2) had no vaginal lavage or medication within 3 days; (3) had no gynecological examination, sexual activity, or vaginal ultrasound within 24 h; (4) were not in the menstrual or pregnancy stages; (5) were not in the acute phase of reproductive tract inflammation; (6) had undergone CDB and ECC simultaneously. The exclusion criteria were as follows: participants who (1) had vaginal lesions; (2) had a history of treatment for cervical lesions (medication, laser, surgery, etc.); (3) had a history of pelvic radiotherapy; (4) had non-diagnostic or unsatisfactory sampling. The flow chart of the screening process is shown in **Figure 1**. This study was approved by the Medical Ethics Committee of the First Affiliated Hospital of Xi’an Jiaotong University, Xi’an, China (Approval Letter No.: XJTU1AF2023LSK-534).

### 2.2 Testing and diagnosis

We used the SINO-ZP24 fully automatic slide preparation system provided by Wuhan Xinuo Intelligent Medicine Co., Ltd. to produce thin layer smears and observe them under a microscope. Cytology results were classified according to the 2014 Bethesda System (8), which includes: negative for intraepithelial lesion or malignancy (NILM), atypical squamous cells of undetermined significance (ASC-US), low-grade squamous intraepithelial lesion (LSIL), high-grade squamous intraepithelial lesion (HSIL), atypical squamous cells that cannot exclude HSIL (ASC-H), squamous cell carcinoma (SCC), atypical glandular cells not otherwise specified (AGC-NOS), atypical glandular cells favoring neoplasia (AGC-FN), adenocarcinoma *in situ* (AIS), and adenocarcinoma of the cervix (AC). This study found no cases of SCC, AIS, or AC. The study population was divided into the following groups: NILM, ASC-US, LSIL, ASC-H, AGC (with fewer cases of AGC-NOS and AGC-FN), and HSIL. A positive cytology result was defined as ASC-US or higher.

The HPV typing test kit provided by Jiangsu Shuoshi Biotechnology Co., Ltd. was used to detect 15 high-risk HPV (HR-HPV) genotypes (16, 18, 31, 33, 35, 39, 45, 51, 52, 53, 56, 58, 59, 66, 68) and 6 low-risk HPV (LR-HPV) genotypes (6, 11, 26, 73, 81, 82). The evaluation and diagnosis results were classified as follows: (1) “HPV positive”: detection of one or more of the above HPV subtypes, categorized into the “HPV16 positive group”, “HPV18 positive group”, “HPV16 and 18 positive group”, “other HR-HPV positive group”, and “LR-HPV positive group”; (2) “HPV negative”: no detection of the above HPV subtypes.

The EDAN-C6HD colposcope, provided by Shenzhen Libang Precision Instrument Co., Ltd., was used to observe the cervix and record the cervical transformation zone (TZ) type (I/II/III), acetowhite changes (none/thin/dense), Lugol staining changes (stained/nonstained), colposcopic impression (benign/LSIL/HSIL/cancer) in patients. The diagnosis was made by two colposcopy professionals. For visible lesions, CDB+ECC was performed. If no significant lesions were observed, a four-quadrant random biopsy+ECC was performed. Biopsy specimens were reviewed by two senior pathologists following the Lower Anogenital Squamous Terminology Standardization Project for HPV-Associated Lesions (9), with categories including: normal, LSIL, HSIL, and invasive cancer. ECC positivity in this study was defined as LSIL or higher lesions.

### 2.3 Data collation and analysis

Data were collected and analyzed using Excel, SPSS Statistics 25.0, and R Studio (4.3.2). *P*<0.05 indicated statistical significance.

#### 2.3.1 Development of the model

The quantitative data in this study did not follow a normal distribution, and the median [interquartile range (IQR)] was used for statistical description. The qualitative data were described using frequencies and rates. The patients’ age, pregnancy and delivery history, menopausal status, HPV status, cytology results, cervical TZ type, acetowhite changes, Lugol staining changes, and colposcopic impression were collected as independent variables. These were analyzed through univariate logistic regression, and significant variables were included in multivariate logistic regression analysis to identify predictive factors for ECC positivity. The “rms” package in R Studio was used to select six predictors and construct a nomogram model. A higher score indicated a greater probability of obtaining a positive ECC result.

#### 2.3.2 Evaluation and validation of models

Calibration evaluates the difference between predicted and actual outcomes. The Hosmer-Lemeshow Test (H-L Test) and calibration curve were used in this study. The calibration curve visually represents the H-L test. The closer the calibration curve is to the standard curve, the better the predictive ability of the nomogram. In the H-L Test, *P*>0.05 indicates good calibration of the model.

The ability of the risk model to distinguish between ECC positive and negative was evaluated using the area under the curve (AUC) of the receiver operating characteristic (ROC) curve. The ROC curve was plotted using the “pROC” package in R Studio, and the AUC value was calculated. An AUC value between 0.5 and 0.6 indicates poor discriminability of the risk prediction model. An AUC value ≥0.75 indicates good discriminability of the risk prediction model.

Clinical utility evaluation assesses the benefit to patients following ECC. The clinical utility of the nomogram model was evaluated using clinical decision curve analysis (DCA), conducted with the “rmda” package in R Studio. If the line corresponding to the threshold probability lies above the “None” and “All” lines, it indicates good clinical utility of the model.

The model validation in this study was internal, focusing on testing repeatability and model fitting during development. The nomogram model was validated using bootstrap sampling (1000 repetitions), where multiple datasets were independently sampled with replacement from the existing data, followed by statistical inference on these new datasets.

## 3 Results

### 3.1 Patient demographic and clinical characteristics

A total of 953 patients were included in this study based on strict inclusion and exclusion criteria. The median age of the study population was 51 years (33, 69), the median number of pregnancies was 3 (2, 4), the median number of deliveries was 2 (1, 2), and 48.90% of the patients were menopausal. Approximately 92.97% (886/953) of patients were HPV-positive, with HPV16 being the most common (38.20%). Cytology-positive cases comprised 51.84% (494/953), with ASC-US and HSIL patients making up the majority at 17.10% and 12.91%, respectively. On colposcopy, acetowhite changes were observed in 52.26%, Lugol staining changes were present in 72.61%, and 59.50% of patients had an LSIL impression. The positive rates of CDB and ECC were 53.09% (506/953) and 31.48% (300/953), respectively. The pathological results of ECC were higher than those of CDB in 48 people (5.04%), as shown in the supplementary table. Univariate logistic regression analysis revealed significant differences in age (*P*<0.001), HPV status (*P*<0.001), cytology results (*P*<0.001), acetowhite changes (*P*<0.001), Lugol staining changes (*P*<0.001), and colposcopic impression (*P*<0.001) between the ECC-positive and ECC-negative groups. However, no statistically significant differences were found in menopausal status, pregnancy and delivery history, or cervical TZ type between the two groups (**Table 1**).

**Table 1:** Demographics and clinical characteristics of study population.

Characteristics	Total *n*	ECC negative	ECC positive	*χ^2^*	*P*-value
		*n* (%)	*n* (%)		
Age (y)				57.631	<0.001
<30	47	31(75.96)	16(34.04)		
30–39	180	143(79.44)	37(20.56)		
40–49	209	147(70.33)	62(29.67)		
50–59	329	177(53.80)	152(46.20)		
≥60	188	155(82.45)	33(17.55)		
Menopausal status				2.224	0.136
Yes	466	330(70.82)	136(29.18)		
No	487	323(66.32)	164(33.68)		
Number of pregnancies				0.562	0.453
0–3	670	464(69.25)	206(30.75)		
4–9	283	189(66.78)	94(33.22)		
Number of deliveries				1.849	0.174
0–2	795	552(69.43)	243(30.57)		
3–7	158	101(63.92)	57(36.08)		
HPV status				29.387	<0.001
Negative	49	40(81.63)	9(18.37)		
LR-HPV+	13	9(69.23)	4(30.77)		
Other HR-HPV+	348	258(74.14)	90(25.86)		
HPV16 and 18+	46	29(63.04)	17(36.96)		
HPV18+	115	90(78.26)	25(21.74)		
HPV16+	364	218(59.89)	146(40.11)		
Unknown	18	9(50.00)	9(50.00)		
Cytology results				63.060	<0.001
NILM	370	290(78.38)	80(21.62)		
ASC-US	163	119(73.01)	44(26.99)		
LSIL	106	61(57.55)	45(42.45)		
ASC-H	91	59(64.84)	32(35.16)		
AGC	11	7(63.64)	4(36.36)		
HSIL	123	51(41.46)	72(58.54)		
Unknown	89	66(74.16)	23(25.84)		
TZ type				4.050	0.132
I	72	50(69.44)	22(30.56)		
II	52	29(55.77)	23(44.23)		
III	829	574(69.24)	255(30.76)		
Acetowhite changes				90.718	<0.001
None	455	370(81.32)	85(18.68)		
Thin	362	233(64.36)	129(35.64)		
Dense	136	50(36.76)	86(63.24)		
Lugol staining changes				47.485	<0.001
Stained	261	225(86.21)	36(13.79)		
Nonstained	692	428(61.85)	246(38.15)		
Colposcopic impression				87.331	<0.001
Benign	192	174(90.63)	18(9.38)		
LSIL	567	397(70.02)	170(29.98)		
HSIL	177	81(45.76)	96(54.24)		
Cancer	17	1(5.88)	16(94.12)		
Total	953	653(68.52)	300(31.48)		

Bold value: *P*<0.05.

### 3.2 Multivariate logistic regression analysis

The significant variables identified in the univariate logistic regression analysis were included in the multivariate logistic regression model. The results indicated that age group, HPV status, cytology results, acetowhite changes, Lugol staining, and colposcopic impression remained significant predictors of ECC positivity. The risk of ECC positivity in women aged 50–59 years was found to be 2.225 (95%CI: 1.086–4.558) times higher than that in women aged <30 years. Compared to patients with negative cytological findings, those with LSIL and HSIL had 2.164 (95%CI: 1.275–3.673) and 2.301 (95%CI: 1.237–4.280) times higher risks of ECC positivity, respectively. Compared to HPV-negative patients, those with HPV16+, HPV16 and 18+, and LR-HPV+ had 2.879 (95%CI: 1.245–6.658), 2.500 (95%CI: 0.872–7.165), and 2.027 (95%CI: 0.428–9.612) times higher risks of ECC positivity, respectively. Patients with a colposcopic impression of LSIL, HSIL, or cancer had 2.188 (95%CI: 1.182–4.049), 2.505 (95%CI: 1.013–6.194), and 41.594 (95%CI: 3.227–536.201) times higher risks of ECC positivity compared to those with a benign impression (**Table 2**).

**Table 2:** Multivariate logistic regression analysis for factors and ECC positivity.

Characteristics	*β*	*Sx*	*Wald*	*P*-value	*OR* (95%*CI*)
Age (y)			57.576	<0.001	
30–39	−0.723	0.392	3.402	0.065	0.485(0.225–1.046)
40–49	−0.237	0.377	0.393	0.531	0.789(0.377–1.654)
50–59	0.800	0.366	4.778	0.029	2.225(1.086–4.558)
≥60	−0.595	0.404	2.177	0.140	0.551(0.250–1.216)
HPV status			19.221	0.004	
LR-HPV+	0.707	0.794	0.792	0.373	2.027(0.428–9.612)
Other HR-HPV+	0.337	0.425	0.628	0.428	1.401(0.609–3.223)
HPV16 and 18+	0.916	0.537	2.910	0.088	2.500(0.872–7.165)
HPV18+	0.336	0.484	0.481	0.488	1.399(0.542–3.611)
HPV16+	1.057	0.428	6.108	0.013	2.879(1.245–6.658)
Unknown	0.783	0.700	1.250	0.264	2.188(0.555–8.631)
Cytology results			14.57	0.024	
ASC-US	0.153	0.246	0.386	0.535	1.165(0.720–1.885)
LSIL	0.772	0.270	8.187	0.004	2.164(1.275–3.673)
ASC-H	0.167	0.319	0.275	0.600	1.182(0.633–2.208)
AGC	−0.841	1.221	0.474	0.491	0.431(0.039–4.721)
HSIL	0.833	0.317	6.929	0.008	2.301(1.237–4.280)
Unknown	0.124	0.318	0.152	0.697	1.132(0.607–2.114)
Acetowhite changes			10.616	0.005	
Thin	0.553	0.206	7.187	0.007	1.739(1.160–2.606)
Dense	1.093	0.367	8.895	0.003	2.984(1.455–6.121)
Lugol staining changes	0.645	0.229	7.917	0.005	1.906(1.216–2.987)
Colposcopic impression			11.691	0.009	
LSIL	0.783	0.314	6.216	0.013	2.188(1.182–4.049)
HSIL	0.918	0.462	3.950	0.047	2.505(1.013–6.194)
Cancer	3.728	1.304	8.168	0.004	41.594(3.227–536.201)
Constant	−3.395	0.608	31.193	<0.001	

Bold value: P<0.05.

### 3.3 Model development

**Figure 2:**
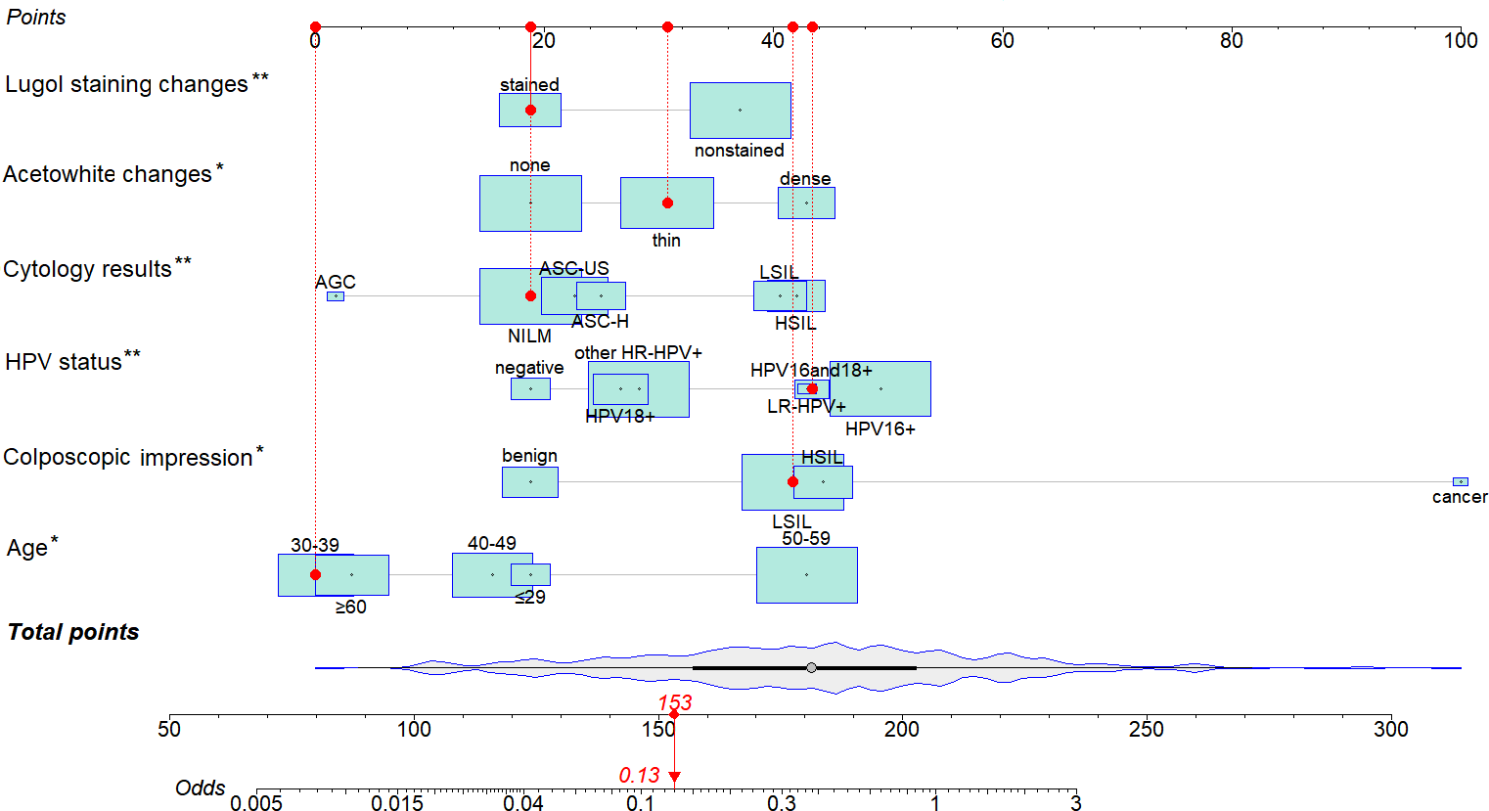
Nomogram for the risk of ECC positivity.

A nomogram prediction model was developed based on six indicators selected through multivariate logistic regression analysis. **Figure 2** is an example of using the nomogram to predict the risk of ECC positivity of a given patient. The patient was 38 years old, with HPV16 and 18+, had a negative cytology result, presented with thin acetowhite and Lugol staining changes, and had a LSIL colposcopic impression. The density plot of total points illustrates the distribution. For categorical variables, their distributions are represented by the size of the box. The importance of each variable was ranked based on the standard deviation along the nomogram scales. To use the nomogram, specific points (red dots) for individual patients are marked on each variable axis. Red lines and dots are drawn upward to determine the points assigned to each variable. The sum of these points (153) is located on the Total Points axis, and a line is drawn downward to the odds axis to determine the risk of ECC positivity (13%).

### 3.4 Model evaluation and validation

**Figure 3:**
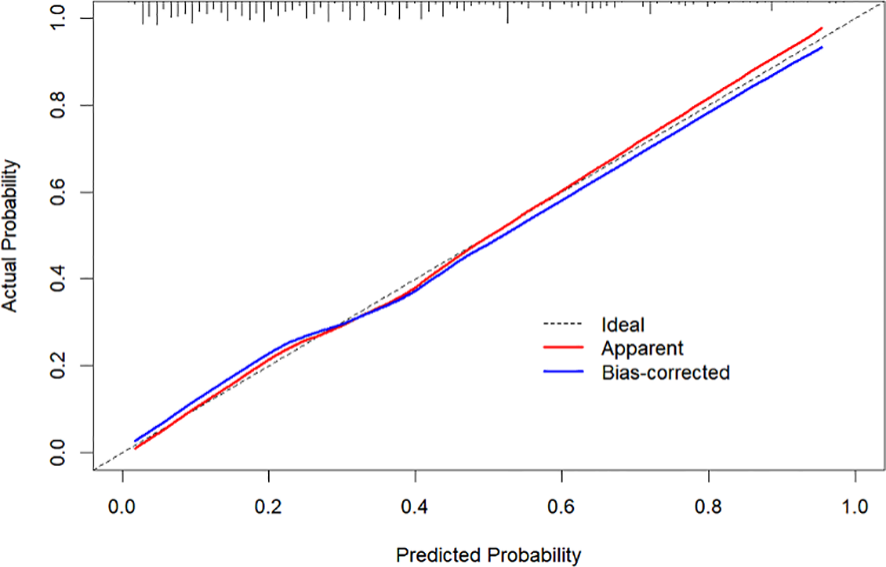
Calibration plot of the model.

The difference between the predicted and actual outcome events was assessed using the calibration graph, as shown in **Figure 3**. The x-axis represents the predicted risk of ECC positivity, while the y-axis represents the actual risk of ECC positivity. The black diagonal dashed line represents the standard curve, and the blue line represents the calibration curve obtained through 1000 bootstrap resampling. A greater overlap between the black diagonal dashed line and the blue line indicates better model performance. The H-L test was performed on the data, yielding a *χ^2^* value of 10.489 (*P*=0.2324), indicating good accuracy of the nomogram model.

**Figure 4:**
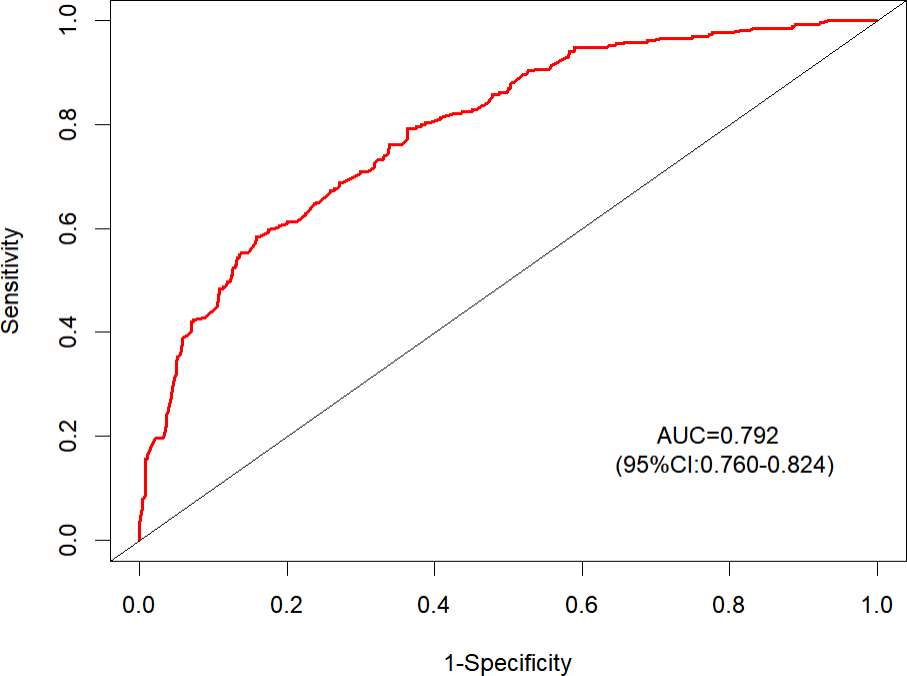
The ROC curve of the model.

The ROC curve of the prediction model is shown in **Figure 4**, with an AUC of 0.792 (95%CI: 0.760–0.824), indicating that the model has good discrimination and can distinguish between outcome and non-outcome events.

**Figure 5:**
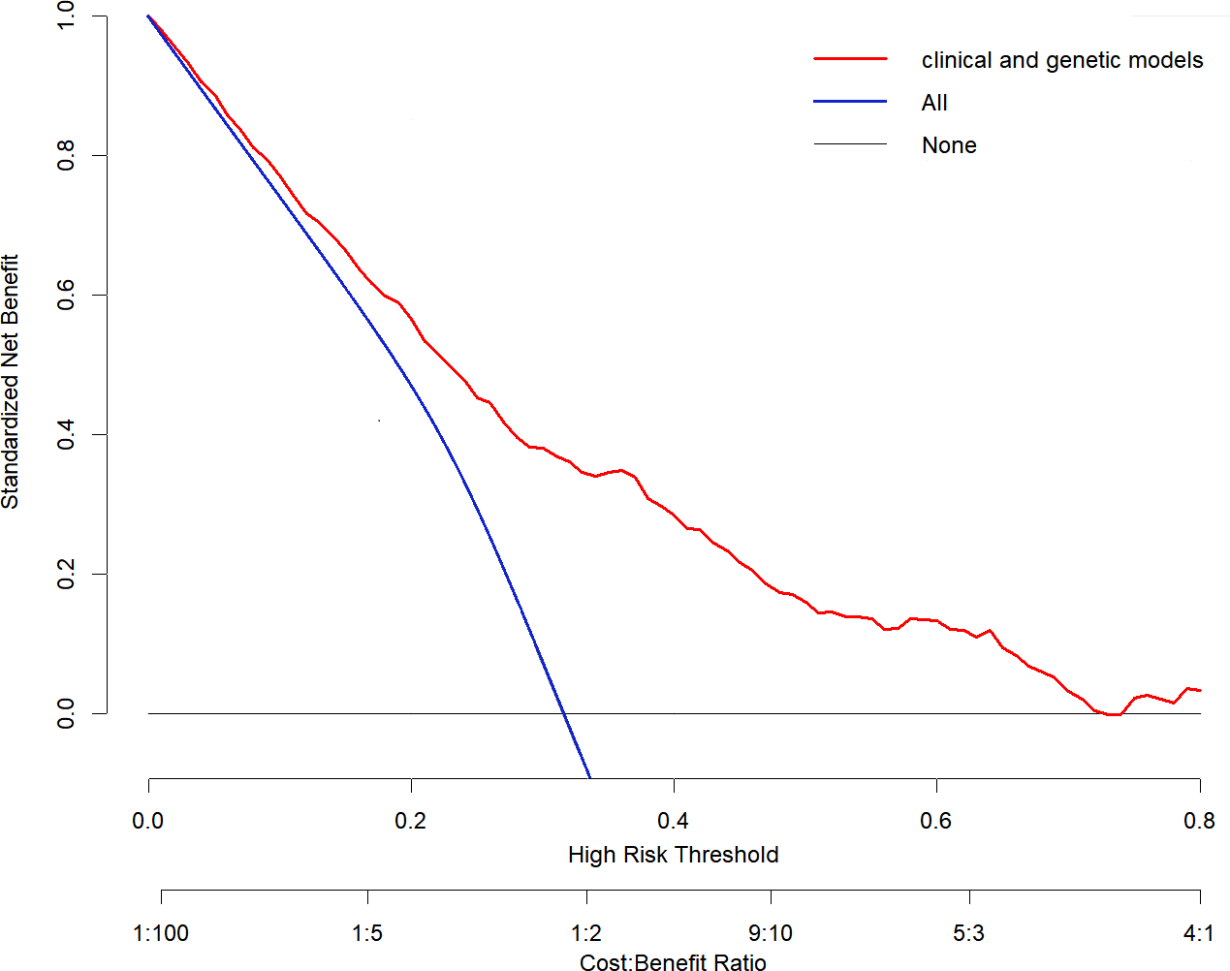
DCA curve of the model.

As shown in **Figure 5**, the DCA curve was used to assess the clinical utility of the model. The “None” line represents the scenario where no positive ECC results are observed in the entire study population, resulting in a net benefit rate of 0. The “All” line represents the scenario where all individuals in the population have positive ECC results and receive clinical intervention. This DCA curve demonstrates that the net benefit rate of the risk prediction model is high, as the red line corresponding to the threshold probability is positioned to the upper right of both the “None” and “All” lines, indicating better clinical utility of the model.

## 4 Discussion

In response to the 2018 WHO call for the elimination of cervical cancer as a public health issue, global expansion of HPV vaccination, regulation of cervical cancer screening, and early treatment of precancerous lesions are essential (1). As part of the histological diagnosis in the “three-step” approach to cervical cancer, ECC aims to reduce missed diagnoses of cervical lesions and prevent treatment delays. However, due to cost-effectiveness concerns, there are no clear recommendations for the indications of ECC in China. In this retrospective study, we explored the factors influencing ECC positivity and developed and validated a nomogram model to assess the risk of ECC positivity.

The nomogram model has been less commonly used in the diagnosis and treatment of cervical lesions, primarily for predicting the prognosis of cervical cancer patients (10, 11). Additionally, prediction models for the risk of ECC positivity have not been widely reported. Many clinicians perform ECC to avoid missing cases of HSIL and higher lesions, even though not all patients may benefit from the procedure. The detection rate of ECC in this study was 31.48% (300/953), which is higher than the rates reported by Zhang et al. and Li et al. (29.18%, 68/233; 27.4%, 450/1638) (12, 13). In clinical practice, physicians focus more on the additional HSIL or higher lesions (“additional yield”) detected by ECC. The detection rate of ECC for HSIL or higher in this study was 17.84%, consistent with the findings of Song et al. (14). However, additional yield of HSIL or higher from ECC was 3.25% in this study, suggesting that 3.25% of HSIL or higher could have been missed otherwise, which is consistent with previous studies (0.6%–5.4%) (14, 15).

In 2023, the ASCCP introduced the ECC Operating Guide (6), recommending ECC for patients who are HPV16+ or HPV18+. This study further categorized HPV results based on clinical application and confirmed that the risk of ECC positivity varies with different HPV types. The study found higher ECC positive rates in patients of HPV16+ (40.11%, 146/364) and HPV16 and 18+ (36.96%, 17/46). In contrast, the ECC positive rate in patients who were HPV18+ was 21.74% (25/115), possibly due to the lower prevalence of HPV18 in this study population. Furthermore, increasing evidence suggests that HPV18 is not the dominant strain among common HPV types in China, with HPV52, HPV58, and HPV53 showing higher prevalence (16, 17). Therefore, the development of ECC-related consensus in China may require more evidence-based medical data from the Chinese population.

The severity of cytological results is also associated with ECC outcomes (18, 19). ECC is currently recommended for patients with cytological results of HSIL, ASC-H, AGC, or cancer (6). This study confirmed the relevance of HSIL and ASC-H results to ECC outcomes. There is ongoing controversy regarding the management of LSIL (20). Studies suggested that most LSIL cases can resolve spontaneously, but 12% of patients may progress to HSIL within 2 years (21). This study also highlighted the risk of positive ECC results in LSIL patients, consistent with the findings of Liu et al. (4). In this study, the ECC positive rate was low in AGC patients, likely due to the small sample size (11 AGC patients in total). Two of these patients had multi-point biopsies for AIS, which may have been limited by the lesion. A comprehensive four-quadrant biopsy might explain the negative ECC results. The sample size needs to be increased to further investigate the characteristics of AGC patients.

In perimenopausal and menopausal women, the SCJ recedes into the endocervical canal with age, and colposcopy cannot be carried out adequately, which increases the importance of ECC in detecting disease and is associated with a higher diagnostic yield in older women (18, 22). Most studies suggested that ECC is more suitable for patients with incompletely visualized of SCJ or type III of TZ (6, 23). According to current German guidelines from the Federal Joint Committee, ECC should be performed for patients with TZ3 when medically indicated (24). However, several researches found no significant correlation between the TZ type and positive ECC results, consistent with this study (4, 13). Additionally, studies have also found that (25), even with SCJ fully visualized, 5.2% of patients still show positive ECC results. And in patients who did not have fully visualized SCJ, no statistically significant association was found between ECC results and final pathological outcomes. This suggested that even when the SCJ is not fully visible, the diagnostic utility of ECC has limitations.

The appropriate age for ECC remains controversial internationally. Solomon et al. found that cervical biopsy sensitivity decreased in women aged over 40, while ECC sensitivity increased (26). An association recommended that ECC is preferred for all women aged older than 40 years (6). Studies have also shown that the risk of HSIL or more severe lesions is 2.653 times higher in women 40–49 years, and 2.545 times higher in women over 50 years, compared to those aged ≤30 years (23). Liu et al. recommended ECC for women aged 45 and older with HPV16+, and for women aged ≥30 years with HSIL or more sever lesions, or with ASC-H, based on an analysis of additional indicators (4). This study further subdivided the age group and validated the risk of ECC positivity in women 50–59 years. Notably, this study found that women aged 60 and over have a low risk of ECC positivity, which aligns with the age distribution of HPV infection and cervical cancer incidence (27, 28). Considering that even though women over 60 may have lower immunity, but they may change in sexual behavior and socio-economic status (25), the underlying reasons need further investigation.

Colposcopic examination is considered an important step in cervical cancer screening and diagnosis, and some studies suggested that ECC should be performed even if colposcopy is satisfactory (SCJ is fully visualized) (29). However, other studies have suggested that routine ECC, when colposcopy is satisfactory, does not significantly improve the diagnosis of low-grade cytological abnormalities with obvious lesions (30). In addition, some studies suggested that colposcopic impression can improve the predictive accuracy of ECC positive results (13, 31). Therefore, this study collected various colposcopic observation items, including the acetowhite changes, Lugol staining, and colposcopic impression. The correlation with ECC positivity was verified, and these items were used as predictors of ECC positivity risk, with different score values assigned for model construction.

This study has several strengths. First, there are few studies on constructing ECC positive risk models, and this study successfully developed and validated a new prediction model, which performed well during validation. Secondly, the prediction model is based on common clinical indicators, with results for each indicator subdivided, offering a clearer reference for clinicians. Nevertheless, this study also has certain limitations. First, as this is a retrospective study, some clinical features are missing or incomplete, such as abnormal vaginal bleeding and postmenopausal duration. Some researchers have suggested that menopause status and the type of TZ are associated with positive ECC results (13, 32), likely due to declining estrogen levels as menopause progresses (33). We considered that exploring the characteristics of the duration of postmenopause could offer innovative insights into ECC-related research. Additionally, due to the limitation of the single-center sample size, this study could not be externally validated in other patient cohorts. Future research will aim to verify and refine the study’s conclusions.

In conclusion, the clinical prediction model developed in this study demonstrates good calibration, differentiation, and clinical utility. It can be used to assess the risk of ECC positivity in patients undergoing colposcopy and provide clinicians with valuable guidance on whether to proceed with further ECC.

## Data Availability

The raw data supporting the conclusions of this article will be made available by the authors, without undue reservation.
