# Supplementary material for: Development and validation of a predictive model for the risk of endocervical curettage positivity
Source: Front Oncol. 2025 Mar 18;15:1559087. doi: 10.3389/fonc.2025.1559087 (PMC11958993; doi:10.3389/fonc.2025.1559087)
Supplement: Supplementary file 1 [file Table1.docx]

Supplementary Material

# Supplementary Table

Pathological diagnosis results of CDB and ECC in 953 patients

| CDB | ECC *n* | | | | Total  *n* |
| --- | --- | --- | --- | --- | --- |
|  | Normal | LSIL | HSIL | Cancer |  |
| Normal | 409 | 17 | 19 | 2 | 447 |
| LSIL | 131 | 104 | 8 | 0 | 243 |
| HSIL | 107 | 9 | 111 | 2 | 229 |
| Cancer | 6 | 0 | 9 | 19 | 34 |
| Total | 653 | 130 | 147 | 23 | 953 |

**
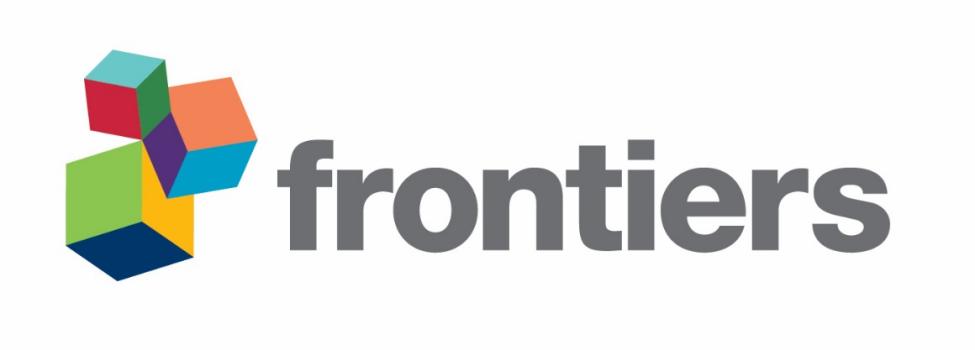
**
